# The Effect of Repeated Blood Harvesting from Pregnant Mares on Haematological Variables

**DOI:** 10.3390/ani14050745

**Published:** 2024-02-28

**Authors:** Charlotta Oddsdóttir, Hanna Kristrún Jónsdóttir, Erla Sturludóttir, Xavier Manteca Vilanova

**Affiliations:** 1 Division of Bacteriology and Pathology, Department of Pathology, Institute for Experimental Pathology at Keldur, Keldnavegi 3, 112 Reykjavík, Iceland; hannakristrun@hi.is; 2 Faculty of Agricultural Sciences, Agricultural University of Iceland, Árleyni 22, 112 Reykjavík, Iceland; erla@lbhi.is; 3 School of Veterinary Science, Universitat Autònoma de Barcelona, 08193 Barcelona, Spain; xavier.manteca@uab.cat

**Keywords:** blood sample, eCG, equine, extensive management, Icelandic horse, pasture, ProCyte DX

## Abstract

**Simple Summary:**

For the extraction of equine chorionic gonadotropin (eCG), mares in the first 2–4 months of pregnancy are harvested for blood. The extracted eCG has been used in pharmaceutical products for the control of reproduction in other animal species. Blood harvesting from mares has raised concerns around their welfare, such as the possibility of them developing anaemia. This study was carried out to investigate the effect on the haematological variables of repeated blood harvesting from pregnant mares in two free-range herds. Our results show that up to 14.3% of mares in one herd had transient moderate to marked anaemia, but that no mares in the other herd developed more than mild anaemia. No mares in either herd had anaemia as defined by Hct three weeks after the last blood harvest. This difference between the herds indicates that external factors such as nutrients ingested by the mares are vitally important in the response to blood loss.

**Abstract:**

Studies have been carried out on the effect of large-volume blood harvesting from horses, but they were performed on nonpregnant horses of various breeds other than Icelandic horses. This study aimed to investigate the effect on the haematological variables indicating erythropoiesis of repeated blood harvesting from pregnant mares. To account for regional variation, two herds of mares were chosen, both kept under free-range conditions. Sequential EDTA blood samples were collected weekly from 160 mares and analysed for haematological variables in an automated analyser. Serum samples from 115 mares were analysed for total protein. In both herds, after three harvests, mares began to measure below the minimum value for erythrocyte numbers, and mild anaemia was present in up to 37% at one time. Mares in only one herd had moderate or marked anaemia, 14.3% of the herd. Both herds showed evidence of increased erythropoiesis, but there was a difference between the herds in the intensity of the response. In both herds, however, all mares had reached the minimum normal haematocrit value three weeks after the last harvest. It is important to investigate the causes for the differences between these herds, which might be addressed to reduce the risk of anaemia.

## 1. Introduction

In Iceland, broodmares are kept under free-range conditions to foal and nurse their foals on pasture. The management of Icelandic horses on pasture is extensive, i.e., horses graze large enclosures with no other feed or supplement through the months of May–November. In some years, a cold spring or early autumnal snowfall will necessitate feeding with wrapped haylage, which is the typical winter feed. Herds are generally given antiparasitic treatment once a year, in the autumn, although some are also treated in the spring. Chosen herds of Icelandic mares in the first 2–4 months of pregnancy are brought in once a week for the harvesting of blood used for the extraction of equine chorionic gonadotropin (eCG), which is found in the circulation during this stage of gestation [1]. The extracted eCG has been used in pharmaceutical products for the control of reproduction in other domestic species, such as cattle [2], pigs [3], sheep [4], and in ovine models for assisted reproductive technologies in humans [5]. Blood harvesting from mares has raised concerns about their welfare, such as the possibility of them developing anaemia. Studies have been carried out on the effect of large-volume blood harvesting from horses [6,7], but those did not include weekly harvesting and were performed on nonpregnant horses of various breeds other than Icelandic horses. In the years 1979–1981, a preliminary study of Icelandic mares harvested for blood over five sequential weeks indicated that haemoglobin and haematocrit were lowest at the third 5 L harvesting, then rose again, and that the values never went outside of the reference values [8]. In recent years, mares have been harvested up to eight times weekly, and it is, therefore, particularly relevant to conduct a study of this kind, and to refer to reference values for pregnant Icelandic mares [9].

It has been shown that haematological values can be influenced by the management type and conditions in which horses are kept [10,11,12]. There is also a variation between breeds, and reference intervals for haematological values in Icelandic riding horses in Austria were found to differ significantly from general reference intervals [13]. A preliminary study on the haematology and clinical chemistry of native riding horses in Iceland also indicated that certain haematological parameters differed from other breeds, and that for some parameters there was possibly an effect from the regional origin of the horses [14]. In assessing the health of horses being harvested for blood, it is most important to evaluate the extent of anaemia and the ability to keep up with blood loss by replacing lost erythrocytes [6]. In horses, haematocrit is widely used for the clinical assessment of anaemia, but clinical reference values have not been defined for cold-blooded breeds, only for hot-blooded horses [15]. The minimum haematological values in cold-blooded horses for red blood cell count is 5.5 × 10^12^/L, and for haemoglobin 80 g/L [16,17]. The minimum normal reference value for haematocrit in cold-blooded horses has been reported as 24% [16] but a scale for assessing the extent of anaemia has not been put forward as it has for hot-blooded breeds [15]. When interpreting haematocrit values, it must also be kept in mind that equine splenic contraction due to stress can lead to a transient increase in the measured values [18]. Only a small proportion of these free-range mares used for harvesting of blood have been trained for riding purposes and the effect of stress due to handling on the haematological results is, therefore, a relevant concern. Signs such as tachypnoea, pawing, whinnying, quivering, sweating, and defecating have been noted as equine behaviours related to stress [19]. Furthermore, physiological responses in connection with large-volume blood collection include sweating, urinating, and defecating during the blood collecting process [7]. To compensate for blood loss, it is important to ensure the availability of microminerals such as iron, copper, and selenium to ensure sufficient haemoglobin synthesis [20,21,22]. Finally, horses that lose whole blood also lose components such as proteins, and it is, therefore, valuable to monitor the resynthesis of the serum proteins lost [16].

As animal welfare assessment relies on a multidimensional approach, the concept can be investigated using different disciplinary approaches. The objective of this study was to investigate the effect on the haematological variables of repeated blood harvesting from pregnant mares, and to focus on this aspect of their welfare. To this end, parameters of erythropoiesis were specially considered. To account for regional variation, two herds of mares were chosen, one in N-Iceland and the other in S-Iceland, both kept under free-range conditions.

## 2. Materials and Methods

The study was carried out under Reg. 460/2017 for the protection of animals used for scientific purposes, as permitted by the Icelandic Food and Veterinary Authority, Ref. No. 2206402.

### 2.1. Animals

Two herds of mares were sampled through the blood harvesting period over 12 weeks in July–October 2022. In a herd in N-Iceland, 66 pregnant mares (whereof 54 were lactating) were sampled, and 103 mares (whereof 83 were lactating) were sampled in a herd in S-Iceland. Stallions had been introduced into the herds in the same week at the beginning of June and extracted again towards the end of July. This ensured that mares in foal were at a maximum of 50 days of gestation at the start of the harvesting period. Mares in both herds had received antiparasitic treatment in October 2021 (The northern herd received Ivermectin subcutaneously and the southern herd was treated perorally with Fenbendazole). Both herds had ad libitum access to grazing of natural pasture on moorland and peatland and had access to mineral lick blocks. At each blood sampling, the mares were assessed for body condition score (BCS) on a scale of 1.0–5.0; with the normal riding horse body condition set at 3.0–3.5 [23]. All horses were kept in free-range conditions, ensuring natural photoperiod and ambient temperature. 

### 2.2. Behaviour Observations

Behaviour of the mares was observed as they entered the stocks for blood harvesting, during harvesting and after they left the stocks. Obvious behavioural patterns consistent with fear or stress were monitored and recorded if noticed before, during and after the blood harvesting of each mare. These signs included sweating, vocalising, pawing, resisting restraint, jumping out of the stocks, or lying down in the stocks, as well as urinating or defecating during the blood harvesting. The scope of this study did not allow for the more time-consuming registration of heart rate and respiration. 

### 2.3. Blood Sampling

Blood samples were taken before each blood harvesting of 5 L, once the area over the jugular vein had been clipped, disinfected and local anaesthesia had been applied (Xylocaine 2%, AstraZeneca, Cambridge, UK). Blood samples were collected into 2 mL EDTA tubes for haematology (Vacuette, Greiner Bio-One, Kremsmünster, Austria) and 4 mL serum tubes with gel separator (Vacuette, Greiner Bio-One, Austria) for total protein analysis. Blood tubes were gently inverted several times to ensure mixing, labelled with the ID number of each mare and transported to the laboratory, where they were kept upright at 4 °C overnight, until analysis was carried out the following day. Serum tubes were allowed to clot, centrifuged at 1932× *g* for 10 min at 4 °C, for serum to be harvested and frozen at −20 °C until analysed.

At each sampling session, blood samples were taken from all mares that were to be harvested for blood on that occasion. The first blood sample was the 0 sample representing the haematological status before any blood had been removed. As all samples were taken prior to each blood harvesting, the subsequent samples represented the status one week after the preceding harvest. Finally, after mares were no longer harvested for blood for that season, samples were collected at two to three occasions subsequently, to obtain samples representing the recovery period of one, two and three weeks after the last harvesting. On each harvesting day, the whole process of blood harvesting and sample collecting from each herd took a maximum of 4 h. Samples were taken by veterinarians and accredited laboratory animal staff.

### 2.4. Haematological and Total Protein Analysis

Haematological values were measured in whole EDTA stabilised blood samples using an IDEXX ProCyte Dx analyser (IDEXX Laboratories, Inc., Westbrook, ME, USA), employing equine-specific, as well as breed and sex-specific settings. Before analysis, samples were allowed to reach room temperature (~20 °C). It was important that all analyses were carried out by the same method throughout the study period; therefore, all samples were kept refrigerated overnight and measured the day after collection. This was to ensure that on the days when maximum numbers of samples were taken, all samples were measured on the same day. A maximum of 30 h elapsed from sampling until haematological analysis was carried out. The analysis focused on variables indicating erythrocyte status, as the main objective was to assess erythropoietic response to blood loss. The following values were analysed: red blood cell count (RBC), haematocrit (Hct), haemoglobin (Hgb), mean corpuscular volume (MCV), mean corpuscular haemoglobin concentration (MCHC), mean corpuscular haemoglobin (MCH), red cell distribution width (RDW).

If indicated, peripheral blood smears were stained with May Grünwald-Giemsa (MGG) quick stain (Bio Optica, Milan, Italy) and examined at ×400 magnification.

Total protein (TP) values were measured in serum using an IDEXX Catalyst One (IDEXX Laboratories, Inc., Maine, USA) analyser, based on the biuret method [24], excluding haemolysed samples to reduce the risk of falsely increased values [25]. Frozen serum samples were brought to room temperature before measurements were performed. Three samples were analysed from each of 115 mares: 0 sample, sample from 3rd or 4th week of blood collection and samples after a recovery period of two or three weeks, depending on sample availability for each mare. Additionally, 0 samples from 17 mares, where second and third samples were not available, were included. Excluded were samples from mares that were harvested less than three times and, in some cases, mares had to be excluded due to haemolysis in one or more samples, since it is known that haemolysis can lead to falsely increased total protein results [26].

### 2.5. Statistical Analysis

To explore if the haematological values changed between the weeks of the blood harvesting period, the method of mixed model was applied [27]. The variables week, age and herd were included in the model as fixed effects and mare as random effect to account for repeated measures within the same individual. The full model was as follows:

y_ijt_ = α_t_ + γ_j_ + (αγ)_jt_ + βa_ij_ + s_ij_ + ϵ_ijt_, (1)

where y_ijt_ is the haematological value for mare i at herd j in week t and a_ij_ is the age of the mare. The α_t_ is the effect of week, γ_j_ is the effect of herd, (αγ)_jt_ is the effect of interaction between weeks and herd, β is the parameter describing the relationship between the haematological variable and age, and s_ij_ is the random effect of mare. The model was fitted with maximum likelihood using the lmer function in the lme4 package [28] in the statistical software R, Version 4.3.1 [29]. Fixed effects were tested with a likelihood ratio test and removed from the model if not significant.

To explore whether the status at recovery (two weeks after the last blood harvesting) could be explained by the number of times the mares went for blood harvesting, a linear regression was conducted.

y_i_ = β_0_ + β_1_w_i_ + β_2_a_i_ + ϵ_i_, (2)

where y_i_ is the haematological value for mare i two weeks after the last blood harvesting, w_i_ is the number of times the mare went for blood harvesting and a_i_ is the age of the mare. This was only tested for the herd in the south as blood was harvested seven or eight times for most of the mares in the other herd.

## 3. Results

### 3.1. Animal Behaviour and Health

The median BCS of the mares in the north was 4.1 (IQR 4–4.2) and in the south the median BCS was 4.0 (IQR 4–4.5). During the 12 weeks with a total of 1337 samples collected, there were 17 registrations of mares in resisting restraint, jumping out of the stocks or lying down in the stocks. Of these, 13 registrations occurred in the southern herd and 4 (whereof two involved the same mare on two separate occasions) in the northern herd. In 4 of these 17 registrations, the exhibited stress coincided with Hct values over the mean, 40.9–48.8% but in most of the mares the Hct values (26.5–36.9%) did not differ from the mean for the respective harvesting session. No counts of sweating, urinating, or defecating in the stocks during harvesting were recorded.

Blood harvesting was discontinued for one mare in the southern herd when a sample from her was measured with an Hct of 16.5% and Hgb of 61 g/L. She also had mild lymphocytosis and monocytosis, which might be indicative of inflammatory disease. A peripheral blood smear revealed hypochromic microcytes among normochromic normocytes. After this, she was monitored weekly by blood analysis and showed recovery throughout the study period. One mare in the northern herd was lame due to a broken hoof and received appropriate treatment by a veterinarian.

### 3.2. Haematological Values

**Figure 1:**
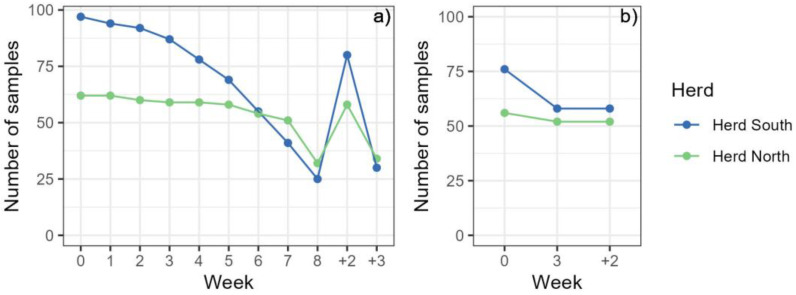
Number of samples (mares) used in the statistical analysis of (**a**) haematological values and (**b**) serum total protein (TP). Samples in week 0 were taken just before the first blood harvesting took place, and then samples were collected weekly during the harvesting season, with the numbers 1–8 representing the number of blood harvesting occasions preceding the analysed sample. The last two sampling points represent two and three weeks after the last blood harvesting (+2, +3). For TP, week 3 comprises samples from either third or fourth harvesting week and week +2 comprises samples from either two or three weeks of recovery.

Mares were harvested for blood on one to nine occasions, determined by the concentration of extractable eCG in the circulation of each mare, with an average frequency of 6.4 times. Notably, a distinction was observed between the two herds: mares in the northern herd exhibited a higher frequency, averaging 7.1 times, compared to the southern herd who averaged 5.9 times. Figure 1 shows a gradual decrease in the number of samples from the southern herd while almost all the mares from the northern herd were harvested seven or eight times. The last sample from the mare who mistakenly was collected nine times was excluded from the statistical analysis because there was just one measurement for that week.

Five mares of unknown age, and four mares that had not been harvested for blood every week, were excluded from the statistical analysis. In total, samples from 160 mares were used in the statistical analysis regarding haematological variables (Figure 1a). For the analysis of TP, for 113 mares, samples taken in week 0, week 3–4 and after 3 weeks of recovery were analysed along with samples taken in week 0 from 23 mares. Five mares of unknown age were excluded from the analysis (Figure 1b). There was only one sample from week 8 (one mare was harvested for the ninth time by mistake) and very few after four weeks of recovery, these samples were therefore excluded from the statistical analysis.

The number and ratio of values below clinical minimum values for erythrocytes at each harvesting were calculated. Clinical minimum values were defined based on reference values for RBC and Hgb, and reference values for Hct in cold-blooded horses derived from clinical reference values for anaemia in hot-blooded horses.

Table 1 shows the descriptive statistics for the analytical results of 1337 blood samples from the two herds, as well as the age range of the 160 mares included in the statistical analysis. Descriptive statistics week by week can be seen in the Supplementary Materials (Table S1).

**Figure 2:**
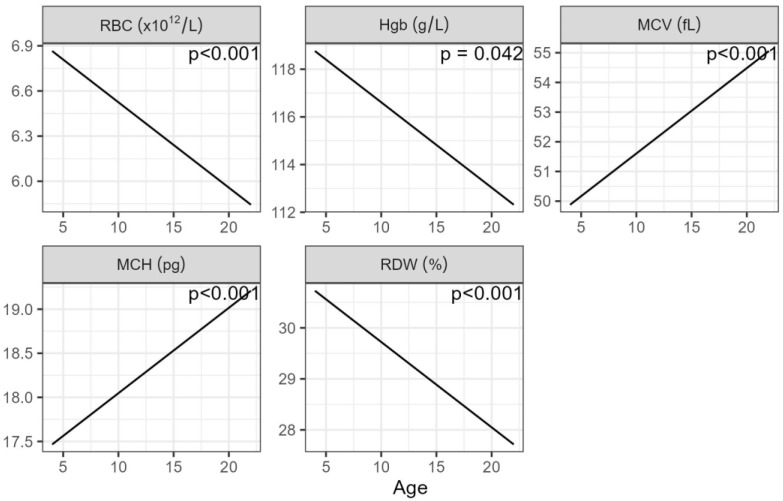
The estimated relationship between the haematological values and age of the mares from the final models (Equation (1)). The p-values are indicative of the significance of the relationship between the haematological values and age. RBC: red blood cell count, Hgb: haemoglobin, MCV: mean corpuscular volume, MCH: mean corpuscular haemoglobin, RDW: red cell distribution width. Age in years.

The age of the mares was included in the statistical analysis to account for the effect of age on the haematological values. Age exhibited a significant inverse correlation with RBC, Hgb, and RDW, while MCV and MCH demonstrated a significant and positive relationship with age (Figure 2).

**Figure 3:**
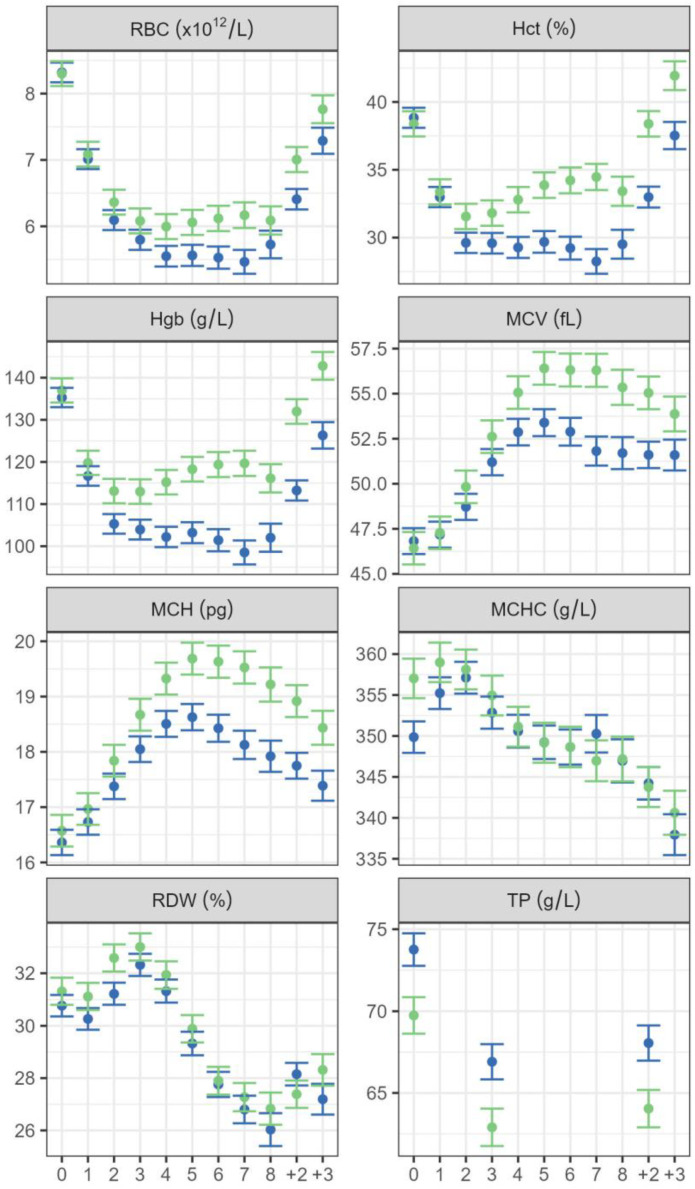
Estimated mean of the haematological variables for average age for the herds in the north (green) and south (blue) from the final model (Equation (1)) for each week of the season, just before the first blood harvesting took place (0), and then weekly during the harvesting season, with the numbers 1–8 representing the number of blood harvesting occasions preceding the analysed sample. The last two sampling points represent two and three weeks after the last blood harvesting (+2, +3). For serum total protein (TP), week 3 represents samples taken one week after the third or fourth harvesting and +2 represents two or three weeks of recovery after the last harvesting. The vertical lines show the 95% confidence intervals. RBC: red blood cell count, Hct: haematocrit, Hgb: haemoglobin, MCV: mean corpuscular volume, MCHC: mean corpuscular haemoglobin concentration, MCH: mean corpuscular haemoglobin, RDW: red cell distribution width, TP: serum total protein.

There was a significant interaction between the week and herd for all the haematological variables (*p* < 0.001 for all variables), indicating that the change with time was different for the two herds. The variables RBC, Hct and Hgb, all showed a similar pattern throughout the period. Values were similar for the two herds before the blood harvesting started, but then decreased during the harvesting season, decreasing more for the southern herd than the northern herd (Figure 3). In the northern herd, the lowest average value of Hgb was 113 g/L and the lowest average Hct value was 32%, both one week after the second and third harvesting sessions. In the southern herd, the lowest average Hgb was between 100 and 105 g/L and the lowest Hct was 29% one week after the second blood harvesting and every week until the blood harvesting was finished. After two and three weeks of recovery, these values were almost back to what they were before the blood harvesting started for both herds.

The erythrocyte variables MCV and MCH showed a similar pattern, with values similar for the two herds before the blood harvesting started but then increasing in both herds with higher values reached in the northern herd. The MCV and MCH value reached the highest values one week after the fifth harvesting in both herds (Figure 3).

The effect of herd was not as obvious for MCHC and RDW as for the other haematological variables. The average values of MCHC and RDW increased when the blood harvesting started but then decreased and remained below the original values at three weeks of recovery (Figure 3). 

**Figure 4:**
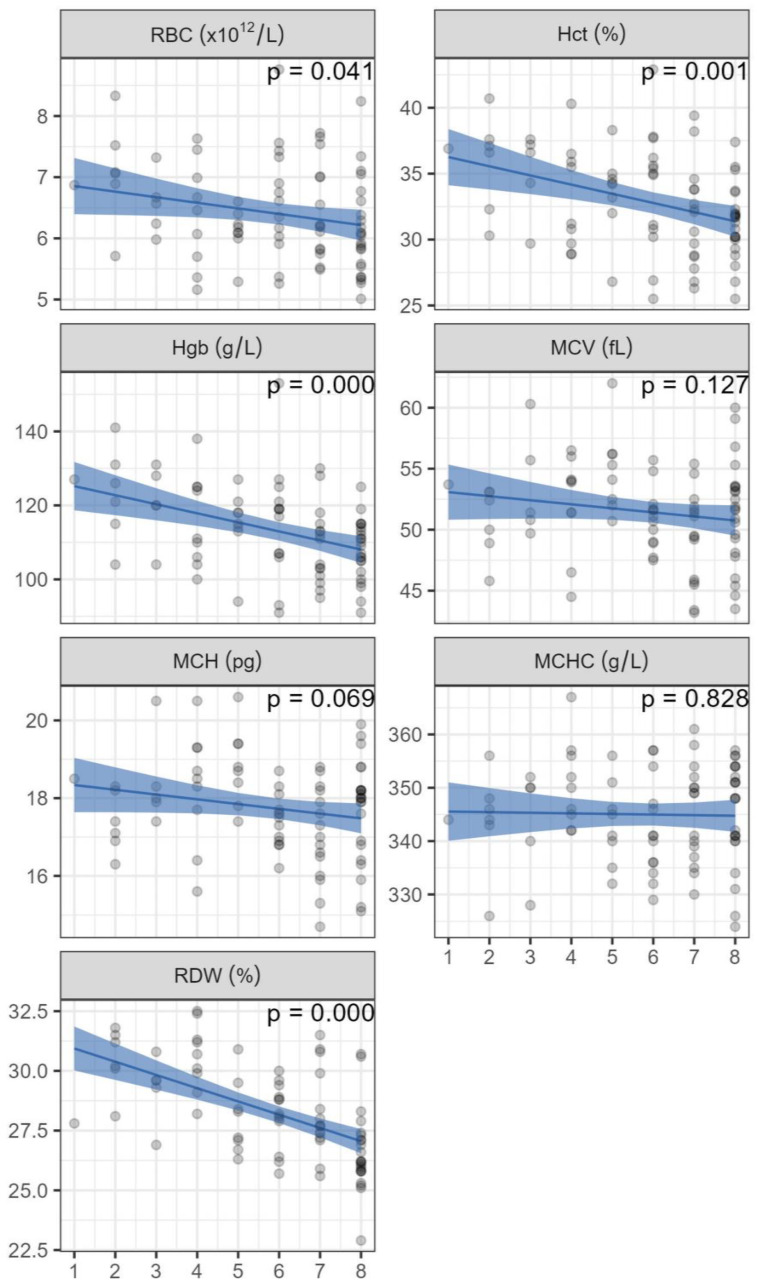
Haematological values (grey dots) for mares in the southern herd, measured two weeks after the final blood harvest (on the y-axis), plotted against the frequency of blood harvesting sessions (on the x-axis). Additionally, a regression line (Equation (2)) using average age, accompanied by a 95% confidence interval, is included. The *p*-values are indicative of the significance of the relationship between the recovery values and the number of blood harvesting sessions. RBC: red blood cell count, Hct: haematocrit, Hgb: haemoglobin, MCV: mean corpuscular volume, MCH: mean corpuscular haemoglobin, MCHC: mean corpuscular haemoglobin concentration, RDW: red cell distribution width.

It was investigated whether the number of weekly blood harvesting sessions the mares went through influenced the subsequent values observed after a two-week recovery period. There was an inverse relationship between the frequency of blood harvesting and the levels of RBC, Hct, Hgb, and RDW (Figure 4). Specifically, mares that underwent blood harvesting four times demonstrated an average Hgb value of 118 g/L and an Hct of 34%, while mares subjected to the procedure eight times exhibited an average Hgb value of 108 g/L and an Hct of 31% following the two-week recovery period.

### 3.3. Total Protein

**Figure 5:**
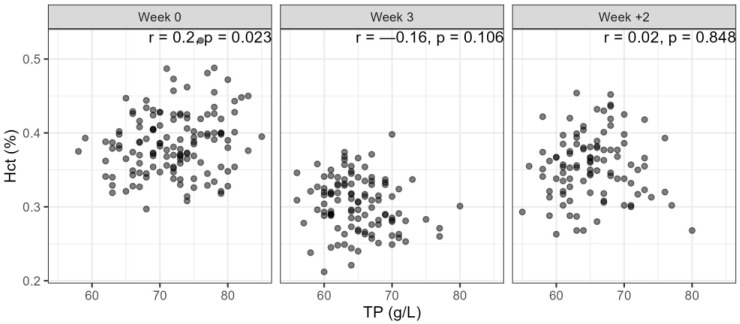
Correlation between haematocrit (Hct) and serum total protein (TP) on three occasions: week (0) just before the first blood harvesting took place, week (3) before the 3rd blood harvesting (3rd or 4th for TP), and week (+2) after two weeks of recovery (two or three weeks of recovery for TP).

Serum total protein values were higher in the herd in the south than in the north (Figure 3). Before the blood harvesting period started, the estimated mean value was 74 g/L for the southern herd while it was 70 g/L in the northern herd. The mean value had dropped by 6.7 g/L after the mares had undergone blood harvesting three or four times and it was similar after the recovery period; however, only four mares measured under 57 g/L, each of them on one occasion. The age of the mares did not have a significant effect on the value of total protein. When analysing the relationship between Hct and TP, there was a weak positive correlation for the 0 samples, but no correlation for week 3 and recovery week 2 (Figure 5).

### 3.4. Minimum Haematological Values

**Figure 6:**
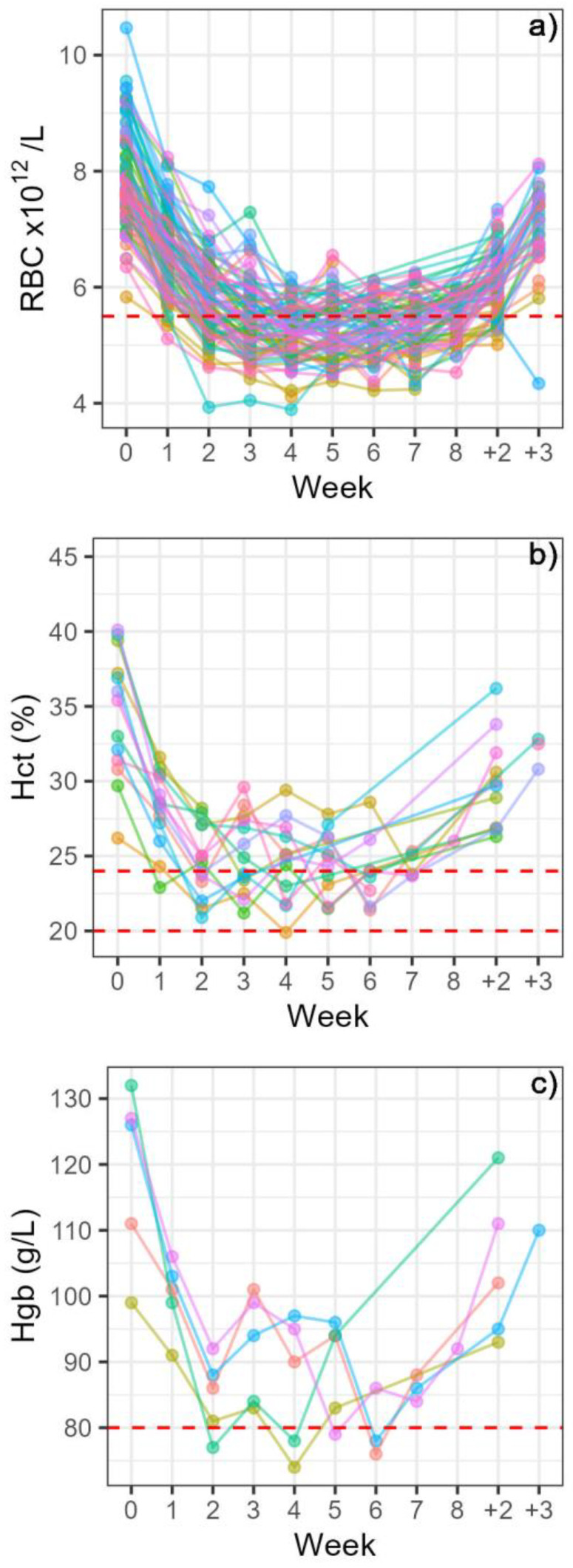
Profiles of the individual mares that at some point measured below reference limits for (**a**) RBC, (**b**) Hct, (**c**) Hgb. Minimum normal reference limits were RBC < 5.5 × 10^12^/L and Hgb < 80 g/L. Clinical reference values for anaemia were 24–26% Hct, mild anaemia; 20–24% Hct, moderate anaemia; Hct < 20%, marked anaemia. Week 0 represents the sampling just before the first blood harvesting, and weeks 1–8 represent the number of blood harvesting occasions preceding the analysed sample. The last two sampling points represent two and three weeks after the last blood harvesting (+2, +3). Each mare is indicated by a colour, with more than one mare being indicated by the same colour in (**b**,**c**), due to the colours being fewer than the mares. RBC: red blood cell count, Hct: haematocrit, Hgb: haemoglobin.

In both herds, there were mares that measured below 5.5 × 10^12^/L for RBC. One week after the second blood harvesting, 26 mares (18% of mares harvested) were under this value and in week seven (one week after the seventh harvesting), 30 mares (48% of mares harvested) were under this value. After two weeks of recovery, 11 mares (8%) were still under this value, and one mare after three weeks of recovery (Figure 6a).

In the northern herd, four mares (6%) had 24–26% Hct in week 2 and four mares (6%) in week 3. In the southern herd, there were 21–37% of mares each week that had 24–26% Hct in weeks 3–7. Only mares in the southern herd measured below 24% Hct, the defined value for moderate anaemia. There were 13 mares that had Hct values under 24% and one mare went under an Hct of 20% corresponding to marked anaemia. Six mares (4% of the southern mares) were under 24% in the second and third week. In other weeks, the proportion was lower (2–4%), and after two weeks of recovery, all the mares were over 24% (Figure 6b). There were five mares that gave a measured Hgb of below 80 g/L at some point, whereof one mare measured below on two occasions, in week two and four. All the mares were above 80 g/L after one week of recovery (Figure 6c).

## 4. Discussion

### 4.1. General Development in Haematological Values

Mean values for Hct were above the minimum reference values throughout the blood harvesting season in both herds. Individual mares in both herds generally showed evidence of a regenerative response of mild to moderate anaemia as indicated by Hct levels 20–26%, and Hgb below 80 g/L. The lowest values in both herds were found one week after two and three weekly blood harvesting occasions, with the herds showing a different pattern in Hct and Hgb development thereafter. The response to blood loss was similar in both herds, characterised by a steady increase in MCV and MCH, reaching a plateau after five blood harvests and remaining high in the three-week period after the last harvesting of blood. The variation in erythrocyte size (RDW) decreased simultaneously and remained low at the end of the study period, confirming that generally, larger erythrocytes were being produced, i.e., macrocytosis was present, but anisocytosis reduced.

### 4.2. Levels of Individual Anaemia

One mare in the southern herd with mild lymphocytosis and monocytosis was likely affected by a general disease, and had marked anaemia after four harvesting sessions, with an Hct of 16.5% and the presence of hypochromic microcytes in a blood smear. Due to her condition, blood harvesting from her was discontinued, and, therefore, she was not included in the statistical analysis of the two herds. This was the only occasion with hypochromic erythrocytes, indicating that in general, mares managed a sufficiently regenerative response. In the northern herd, 6% of mares had mild anaemia in weeks 2 and 3, and in the southern herd, 19–37% of mares had mild anaemia during weeks 3–7. Only one mare was under 26% Hct after two weeks of recovery, with an Hct of 25.5% and all mares had an Hct of ≥26% after 3 weeks of recovery. In the 160 mares included in the statistical analysis, one mare had an Hct under 20% on one occasion, corresponding to marked anaemia, and 13 mares had moderate anaemia with Hct between 20 and 24% on one or more occasions. All these mares were in the southern herd, and thus 14.3% of mares in that herd exhibited moderate to marked anaemia, whereas no mare in the northern herd had an Hct below 24%. The lowest mean Hct in the southern herd was 27%, seen one week after the seventh harvesting, whereas in the northern herd the lowest mean of 32% was seen one week after the second and third harvesting occasions. All mean values were, therefore, above the limits for anaemia in cold-blooded horses. Haematocrit is the value most used to assess severity of equine anaemia, and a scale has been defined for hot-blooded breeds: mild anaemia is defined as Hct 30–34%, moderate anaemia as 20–30% Hct and marked anaemia as Hct < 20% [15,30]. The minimum Hct normal reference value for cold-blooded breeds such as the Icelandic horse is lower, or 24% [16]. Clinically healthy native Icelandic riding horses have been found with an Hct reference interval of 25–39% [14] and healthy pregnant mares had the lowest measured value of 26% [9]. However, as our study did not allow thorough clinical examination, and to reduce the risk of underestimating anaemia regarding Hct values, the scale used in our study was as follows: mild anaemia, 24–26%; moderate anaemia, 20–24%; marked anaemia under 20% [15,16]. Minimum reference values for RBC and Hgb in the cold-blooded breeds were used for the indication of mild anaemia, as these variables are less used and have not been defined for the clinical diagnosis of anaemia. To give an idea of the critical levels of Hct in a clinical setting, a value of between 15 and 20% is the recommended target when administering blood products in response to haemorrhage [31].

In our study, each haematological analysis represents the status one week after the preceding blood harvesting, making possible the assessment of the pre-harvesting status of each mare each week. Our results indicate that three weeks after the last harvesting occasion, all but one mare were over the minimum RBC value of 5.5 × 10^12^/L and all mares had an Hct of 26% or higher, and Hgb of over 80 g/L. The mares in the northern herd even reached the values found before blood harvesting began. Previous studies have indicated that, after reaching their lowest point one week after the harvesting of 8 L of blood, these variables were found to rise during the subsequent two weeks [6]. Repeating the 8 L harvesting every three weeks for a total of five harvesting sessions, these variables had reached higher values immediately prior to each harvest, and at the fourth harvest (week nine), they were comparable to the values at the beginning [6]. Other reports have reported the complete restoration of red cell variables to pre-harvest values taking from 26 to 84 days, but these studies involved the removal of a much larger proportion of blood than our study, 9 L daily for three consecutive days [32] and 8 L weekly for eight weeks from light horses and ponies [33]. The Icelandic horse measures around 140 cm at the withers and a horse in riding body condition weighs on average 366 kg [34,35]. The mares in this study were not in riding condition as they generally had a BCS of or above 4 out of 5 and therefore would likely weigh around or over 428 kg [35]. The herds are kept and managed by their owners in pastures where there are no other facilities than holding pens, so weighing the animals was not an option. It would have added to the results if data on weight were available. However, the aim of the study was to assess the ability of the mares to respond to repeated blood loss; therefore, we still feel that the results do address the research question.

An important influence on haematocrit in horses is the spleen and it should be borne in mind that our results could to some extent be influenced by splenic contraction, possibly masking the reduction in haematocrit. It has been demonstrated that splenic contraction in horses under low-intensity exercise can increase Hct by at least 10%, and that the equine spleen is also a reservoir for plasma [36]. It has also been shown that after 30 min of exercise at 5 m/s, the mean Hct increased from 28.5 to 41.3% [18]. Over six litres of erythrocyte-rich blood are temporarily sequestered in the spleen and can be directed to the bloodstream and recalled to the spleen as needed [37]. As this study involved sequential samples from each mare, the development throughout the study period was expected to reveal over time if the splenic reserve was not managing to compensate the blood loss. Our study showed that mares undergoing blood harvesting eight times had lower average Hgb and Hct values following the two-week recovery period than those harvested four times. It is, therefore, clear that even though reference intervals were reached within two weeks after the last harvesting session, increased numbers of harvesting sessions might lead to a longer recovery period. It could be inferred that with increased numbers of harvesting, the splenic reserve can respond less effectively to blood loss. In assessing the extent of the influence of the spleen, its weight seems to be independent of the weight of the individual horse, and rather is correlated to the total calculated erythrocyte volume [38]. In splenectomised horses, it was shown that a certain excess of erythrocytes is kept as an oxygen-carrying reserve, and that maximal oxygen demand, rather than basal metabolic needs, determines the total erythrocyte mass [38]. This maximal oxygen demand is also why reference intervals for Hct are different for hot-blooded and cold-blooded breeds. 

Most of the mares in the study were accustomed to the procedure of blood harvesting and there were few occasions of overt stress response. Four of the mares who showed signs of stress in this study had increased Hct values when compared with the mean, but this was not a constant factor for those mares between weeks. The younger, less habituated mares did not show a tendency to higher Hct when compared to the mean. The mares in this study were not under athletic pressure, herded calmly on a weekly interval from the pasture to the pen, and thus would not have the same needs for a total erythrocyte mass as a Thoroughbred racehorse; therefore, it is likely that their splenic reserve is in the lower range of that expected in horses.

### 4.3. Compensation for Blood Loss

In the present study, MCV and MCH were still elevated three weeks after the last blood harvest even though RBC, Hct and Hgb had reached normal levels. Horses that lose considerable amounts of blood will compensate using physiological adjustments and regeneration of blood components [39]. The mean volume of erythrocytes (MCV) will increase when higher numbers of larger, less mature cells are released from the bone marrow [17]. In a study where horse blood was harvested at 16 mL/kg BW at three-weekly-intervals over a 12-week period, a sustained increase in MCV was seen throughout the study period even though recovery was reached for Hgb, RBC and Hct after each harvesting [6]. In an experiment involving induced anaemia, MCV values remained increased after RBC and Hct values returned to pre-anaemia control levels [40]. Therefore, increased MCV values in the presence of other normal parameters suggest that the horse may recently have gone through regenerative anaemia. In addition to immature cells being larger, they have a slightly reduced lifespan [32] and, therefore, it can be expected that as blood loss continues, the demand for newly produced erythrocytes increases. A reduction in erythrocyte numbers will also be counteracted by a higher concentration of haemoglobin and MCH and MCHC increase to enable more efficient oxygen binding [6]. 

In the present study, the haemoglobin in five mares in the southern herd measured below 80 g/dL, one of them twice, whereas this was not true of any mare in the northern herd, where the lowest value was 88 g/dL in two mares. In general, mares were able to increase erythrocyte size and haemoglobin concentration to counteract the loss of erythrocytes, indicating that iron supply was adequate [6]. Even so, one mare, did exhibit traits of iron-deficiency anaemia, with the appearance of hypochromic microcytes, which otherwise were not a feature. In past years, mares being harvested for blood were monitored by analysing haemoglobin values, and in our study Hct and Hgb values were highly positively correlated (r = 0.98). The same was true for RBC values, which were positively correlated with Hct (r = 0.83). Mares which were under 24% Hct had Hgb values of 74–88 g/L and RBC values of 3.9–6.0. Therefore, measuring only Hgb could lead to a few, but likely not many, mildly anaemic, mares being undetected.

It should be kept in mind that iron deficiency generally has a negligible effect on haemoglobin production in horses [33,41]. However, the difference between the two herds in recovery rate for erythrocyte values indicates that extrinsic factors such as nutrients in feed can have an influence on erythropoiesis. Such differences in the haematological values between regions in Iceland have been seen before, as calculated reference values differed between riding horses in S-Iceland, and horses in N-Iceland and the capital area [18]. In our study, the herd that reached lower values and seemed to take longer to recover, grazed in a region where iron-rich ash fell in the Eyjafjallajökull eruption in 2010 [42], even though information is lacking on the effects to this day on pasture mineral composition. It is possible that the mares feeding on grasses grown in iron-rich soil will be in a state of inhibited absorption of iron to reduce the risk of iron overload, for example, due to intestinal mucosal block [20]. However, the availability of iron in feed is not the only factor, with other microelements of importance, such as copper and selenium [43,44]. This difference between the herds warrants further investigation into the correlation between nutrition and haematological dynamics and indicates the importance of focusing on the nutrition of horses being harvested for blood. Mares in both herds originate from herds that have been harvested for blood for several generations, and some of them have direct relatives in the other herd. Therefore, a familial or genetic factor is not suspected to influence the differences between the herds.

Horses differ from other species in terms of erythropoiesis and erythrocyte maturation, with much of the final maturation taking place in the spleen; reticulocytes are rarely encountered in equine blood [15,45]. The diagnosis and categorisation of equine anaemia is, therefore, subject to different factors than those of many other species. In clinical samples from anaemic horses of various breeds, a reticulocyte increase was not seen unless Hct was under 20% [15], and in a study where blood loss anaemia was induced in horses, circulating reticulocytes were not found until the haematocrit had reached and remained at 13–14% for several days [46]. Also, reticulocytes are significantly fewer in blood samples from cold-blooded than from warm- and hot-blooded breeds [15]. It is, therefore, highly unlikely that increased numbers of reticulocytes could have been identified in our study. Automated analysers have been inconsistent in the identification of equine reticulocytes [47], and the ProCyte Dx does not include them under the equine settings.

In assessing the effect of blood loss on a horse, the rate of blood loss must be considered. After acute haemorrhage, it will take up to 48 h for RBC, Hct and Hgb to reach the lowest point [31,48]. Total protein will also fall below normal values but will start to recover after about 24 h [48]. In the case of chronic or slow, steady blood loss, the resulting anaemia develops slowly, and due to physiological adaptions, Hct can reach low levels before clinical signs become obvious [49]. Blood-loss anaemia becomes less regenerative with time due to loss of nutrients like iron and protein [50]. A regenerative response does take place but might be less intense than with acute blood loss, hypoproteinaemia is usually observed, and microcytosis and hypochromatosis, indicative of iron deficiency anaemia, develop over time as iron stores are depleted [49]. Weekly harvesting of blood could be described as repeated acute blood loss. In our study, serum total protein generally measured higher in the southern herd, and only five mares measured under the reference minimum of 58 g/dL for Icelandic horses [51] in the samples taken during the harvesting and during recovery. The lack of correlation between TP and Hct values indicated that it is unlikely that dehydration or splenic masking of anaemia were a general feature in the mares. In the hours after blood loss, the spleen can mask changes in Hct, and it may take one to two days for the Hct to reflect the true severity of the anaemia [50].

It was interesting that mean erythrocyte numbers and haemoglobin decreased with increasing age. One study reported an increase in haemoglobin levels [52] while another one reported a decline, although without statistical significance [53]. A decrease in erythrocyte numbers associated with ageing seems to be a frequent finding, along with an increase in MCV and MCH [51,52,53,54]. In the statistical analysis, a separation into age groups for each herd resulted in groups containing few individuals (Supplementary File S1, Table S2). The graphical depiction of this analysis is included as Supplementary Material (Supplementary File S1, Figure S1), but in this presentation, the difference between herds is less obvious; therefore, it was preferred not to present this as the main figure. A closer look at the relationship between age and haematological values (RBC, Hgb, MCV and MCH) indicated that the relationship was stronger before the blood harvesting took place (week 0) than when blood had been harvested several times. Therefore, as erythropoiesis increased in the younger mares and compensatory mechanisms were activated, they veered towards fewer, larger erythrocytes. The fact that there was less difference with age as the harvesting went on illustrates that the older mares were no less capable of reacting to blood loss.

### 4.4. Recovery after Last Blood Harvest

When three weeks had passed since blood was last harvested, no mares in this study were anaemic according to RBC, Hct and Hgb values, but MCV and MCH were still elevated. This is in accordance with previous research showing that the erythrocyte regenerative response in horses is slower than in some other domestic species and might take 4–12 weeks to regain base levels [55]. As previously mentioned, increased MCV is a more sensitive indicator of regeneration than changes in haematocrit, being the first and most consistent parameter to show a significant change following the development of anaemia [40]. To identify maximally stimulated erythropoiesis in routine haematology, the best indicators are anisocytosis and increased MCV by up to 10 to 15 fL above baseline levels for an individual horse [56]. In a study where anaemia was induced in four horses by harvesting 6L three times a week until a 2% peripheral reticulocytosis was achieved, MCV increased from the average baseline value of 45 fL to 66.5 fL in response to the blood loss [46]. In our study, ten mares in the southern herd and 33 mares in the northern herd increased by 10–15 fL, with two northern mares even reaching up to 16 fL over baseline values. This difference further indicates that mares in the northern herd showed a more intense and effective regenerative response to blood loss than the southern mares.

### 4.5. Limitations of This Study

In this study, we investigated weekly the haematological status of mares preceding blood harvesting. This was carried out by utilizing the weekly herding of mares and their foals, and the intent was not to disturb the animals more than already had occurred. Therefore, even though it would be interesting to follow the immediate response to blood removal, it was not possible to take samples during the hours and days after each harvesting. This is also outside of the scope of this study. There were also limits that apply to the collection of data at each harvesting. It was necessary to utilise each harvesting session well, without delaying the process or causing stress to the animals. It was, therefore, not possible to carry out time-consuming clinical assessment and behavioural analysis that would have given valuable insight into the physiological effect of blood harvesting on the mares. However, it was important to ensure the uniformity and representativeness of the blood samples. All overt behavioural patterns indicating stress were noted, as well as the general demeanour and indications of health.

A difference could be expected in haematological values between lactating and non-lactating mares. However, in both herds, by far most mares were lactating, and, therefore, the non-lactating group was not large enough for statistical significance. A significant three-way interaction was found for RBC (Figure S2) but this relationship was difficult to interpret. For RBC and Hct in weeks 0 and 1, the tendency was for the lactating mares to have lower values, previously hypothesised to represent haemodilution in relation to lactation [57]. For the remainder of the blood harvesting, this difference is not notable between the groups.

To ensure uniform handling of samples, the study was planned to consider the maximum number of samples that would need analysing at the same time. The samples were always taken on the same day of the week and analysed the next day, after being kept at 4 °C overnight. Therefore, all samples were treated in the same way and so were suitable for haematological analysis and comparable between sampling dates [58]. This method of sample collection and handling reduced the reliability of platelet values [59]. Platelet assessment is a valuable tool in evaluating the response to blood loss [60]. However, the availability of EDTA tubes and refrigeration of samples were deemed important to ensure the reliability and comparability of other values. Visual inspection of blood smears confirmed that platelet aggregation gave rise to a change in the platelet values and, therefore, it was decided to exclude platelet values from the analysis. For the same reason of prioritising and time-limits, smears were not carried out consistently, but were performed when haematological analysis indicated that inspection of cells was needed, such as the abovementioned platelet inconsistencies, the suspicion of marked anaemia and leukocyte counts outside reference intervals.

Total protein analysis was not carried out on all sequential samples, due to financial and time constraints. However, it was decided that it was valuable as an aid to assessing the hydration status and splenic influence on the results of Hct. The sampling points chosen for TP analysis were (a) the 0 sample, (b) the point of lowest Hct mean values and (c) the last point during recovery period. The information gained from these three points were helpful in the interpretation of other results.

## 5. Conclusions

This study shows that the weekly removal of around 5 L of blood led to moderate to marked regenerative anaemia in up to 14.3% of mares in one herd, but that no mares in the other herd developed more than mild anaemia. Obvious behavioural indications of stress were noted in 1.3% of cases of blood harvesting (17/1337 cases) but were likely to have influenced Hct values in only four of these. Mares in both herds showed a compensatory response of erythropoiesis and no mares had anaemia as defined by Hct three weeks after the last blood harvest. It could be concluded that the effect of blood harvesting was generally mild and of short duration. Samples taken three weeks after the last blood harvest showed that there was still evidence of erythropoiesis, as indicated by MCV and MCH. Mares that were harvested four times showed slightly higher recovery values of Hct and Hgb than mares that were harvested eight times. There was a difference between the two herds in the effectiveness of their erythropoietic responses. This difference in the development of anaemia and the compensative response to blood loss warrants further research into the processes of erythropoiesis. This should include the analysis of the mineral content and ratios of the pastures where the two herds graze.

## Figures and Tables

**Table 1:** Descriptive statistics for the blood variables and age in 160 mares harvested for blood.

Variable	Unit	N	Min	Mean	SD	Max
RBC	×10^12^/L	1337	3.89	6.43	1.16	11.44
Hct	%	1337	20	33	5.1	53
Hgb	g/L	1337	74	115	16	179
MCV	fL	1337	36.8	51.5	5	66.5
MCH	Pg	1337	13.5	18.1	1.6	22.6
MCHC	g/L	1337	308	351	11	384
RDW	%	1337	22.9	30	2.9	39.8
TP	g/L	362	55	68	6.1	85
Age	Years	160	4	11	4.4	22

Analysis of blood samples from pregnant Icelandic mares subjected to weekly blood harvesting. Erythrocyte variables (RBC, Hct, Hgb, MCV, MCH, MCHC, RDW) in blood samples measured in 160 mares weekly during the blood harvesting period, 2022. Samples for haematology were 1337 in total. Serum total protein (TP) was analysed in 362 samples from 132 mares. The table also shows descriptive statistics for the age of the mares. N is the total number of samples for the analysed blood variables, and number of mares for the age. RBC: red blood cell count, Hct: haematocrit, Hgb: haemoglobin, MCV: mean corpuscular volume, MCHC: mean corpuscular haemoglobin concentration, MCH: mean corpuscular haemoglobin, RDW: red cell distribution width, TP: serum total protein.

## Data Availability

The original contributions presented in the study are included in the article/Supplementary Materials; further inquiries can be directed to the corresponding author/s.

## Supplementary Materials

The following supporting information can be downloaded at: https://www.mdpi.com/article/10.3390/ani14050745/s1, Table S1: Descriptive statistics for the haematological variables by week. Supplementary File S1: Effect of age groups. Table S2: Number of mares in each age group in each herd; Figure S1: Estimated mean of the haematological variables for each herd and age-group. Supplementary File S2: Figure S2. Estimated mean of the haematological variables for each herd, lactation status and average age.
