# Supplementary material for: The Effect of Repeated Blood Harvesting from Pregnant Mares on Haematological Variables
Source: Animals (Basel). 2024 Feb 28;14(5):745. doi: 10.3390/ani14050745 (PMC10931342; doi:10.3390/ani14050745)
Supplement: Supplementary file 1 [file animals-14-00745-s001.zip › animals-2867686 Supplementary File S1.pdf]

File S1 – Effect of age groups

Introduction

It is known that age can influence haematological values of horses [52-55]. It is possible to analyse the age effect by assuming linear relationship and treat age as a continuous variable. It is also possible to divide the mares into age groups to test the effect of age and also to analyse if the effects of others variable such as herd or number of weeks blood has been harvested depend on the age group.

Statistical analysis

To explore if the haematological values changed between weeks of the blood harvesting period and to test the effect of age the method of mixed model was applied [27]. The variables week, age and herd were included in the model as fixed effects and mare as random effect to account for repeated measures within the same individual. The full model was as follows:

$$y_{ijkt} = \alpha_t + \gamma_j + \beta_k + (\alpha\gamma)_{jt} + (\alpha\beta)_{jk} + (\gamma\beta)_{jt} + (\alpha\gamma\beta)_{jkt} + s_{ij} + \epsilon_{ijkt} \quad \text{(Eq. S1)}$$

where y_{ijt} is the haematological value for mare *i* at herd *j* in week *t* and in age group *k*. The α_t is the effect of week, γ_j is the effect of herd and β_k is the effect of age group. Interactions between the main terms were included in the model to account for that effects of one variable could depend on another variable The term s_{ij} is the random effect of mare. The model was fitted with maximum likelihood using the lmer function in the lme4 package [28] in the statistical software R [29]. Fixed effects were tested with a likelihood ratio test and removed from the model if not significant.

Results

The mares were divided into three age groups (Table S2) and an analysis was carried out to test the effect of age, herd, and the effects of numbers of blood harvesting weeks on haematological variables.

Table S2. Number of mares in each age group in each herd.

Herd	Age group	N
South	4-10	43
	11-15	39
	15-22	16
North	4-10	20
	11-15	30
	15-22	12

The results showed that for RBC the interaction between age group and week was significant ($p<0.001$) as well as the interaction between herd and week ($p<0.001$). This indicates that the change in the estimated mean value of RBC between weeks was not the same for all age groups. The difference between age groups was larger in week 0 than in later weeks (Figure S1). For Hct, Hgb, MCV and MCH the interaction between herd and week was significant ($p<0.001$ for all variables) and the effect of age group was also significant ($p=0.014$, $p=0.011$, $p<0.001$, $p<0.001$, respectively). For MCHC the interaction between herd and week was significant ($p<0.001$) but the effect of age was not significant ($p=0.377$). The interactions between age group and week was significant ($p=0.035$) as well as the interaction between herd and week ($p<0.001$) for RDW.

The younger age groups had higher estimated means of RBC, Hct and Hgb than the older age group. The youngest age group had lower estimated means of MCV and MCH than the older two groups and the estimated mean of RDW was higher for the youngest group than the older ones. There was no effect of age on MCHC (Figure S1).

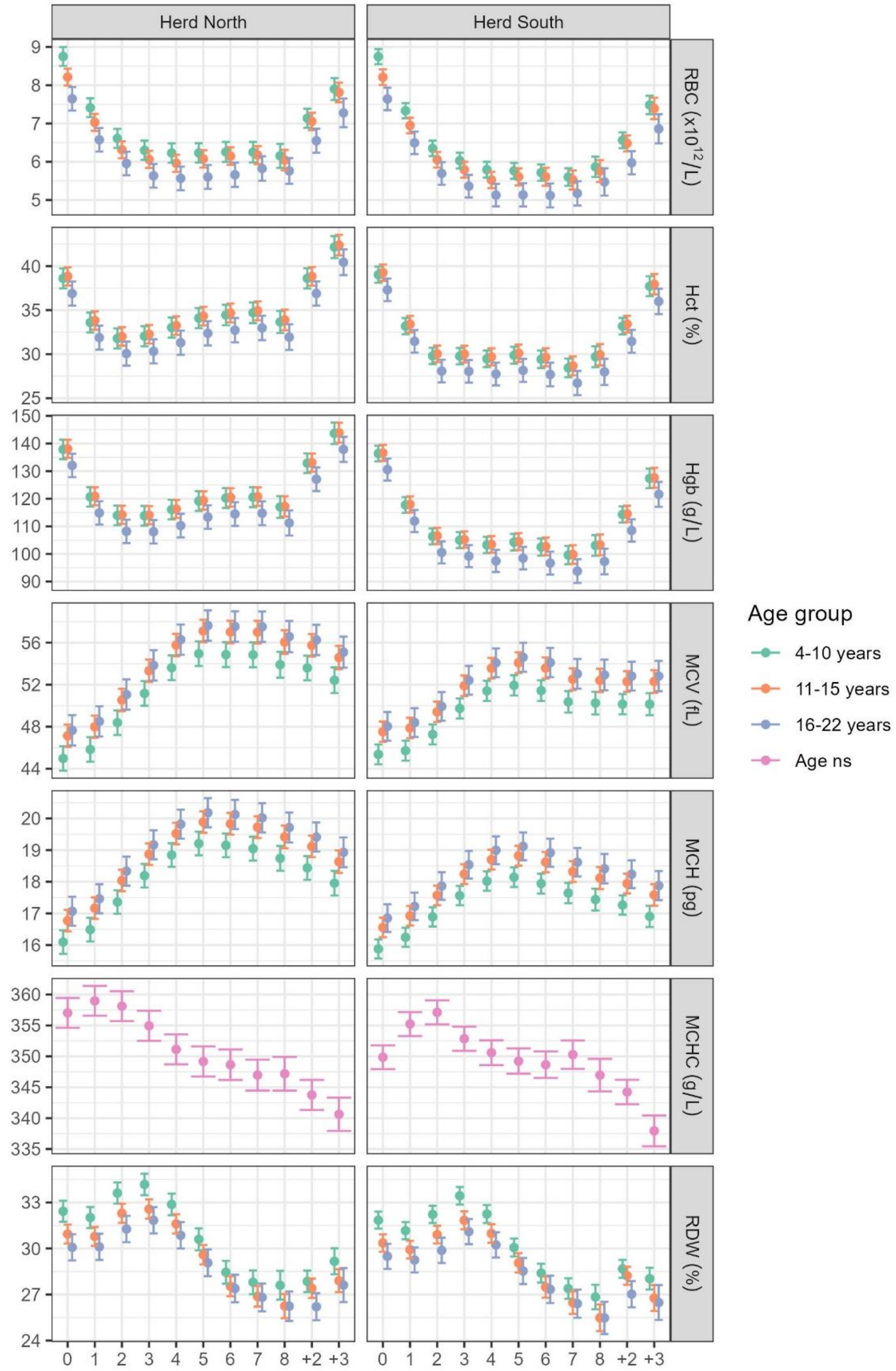

Figure S1. Estimated mean of the haematological variables for each herd and age-group. From the final models (Eq. S2) for each week of the season: just before the first blood harvesting took place (0), and then weekly during the harvesting season, with the numbers 1-8 representing the number of blood harvesting occasions preceding the analysed sample. The last two sampling points represent two and three weeks after the last blood harvesting (+2,+3). The vertical lines show the 95% confidence intervals. RBC: red blood cell count, Hct: haematocrit, Hgb: haemoglobin, MCV: Mean corpuscular volume, MCHC: mean corpuscular haemoglobin concentration, MCH: mean corpuscular haemoglobin, RDW: red cell distribution width.
