# Supplementary material for: The Effect of Repeated Blood Harvesting from Pregnant Mares on Haematological Variables
Source: Animals (Basel). 2024 Feb 28;14(5):745. doi: 10.3390/ani14050745 (PMC10931342; doi:10.3390/ani14050745)
Supplement: Supplementary file 1 [file animals-14-00745-s001.zip › animals-2867686 Supplementary File S2.pdf]

File S2 – Effect of lactation status

Introduction

Whether mares are lactating, or non-lactating could have an impact on how they respond to repeated blood loss that takes place when their blood is harvested for equine chorionic gonadotropin. There were not many mares that were non-lactating. The results from the analysis on the effects of the lactation status should therefore be interpreted with caution.

Statistical analysis

To explore if the haematological values changed between weeks of the blood harvesting period and to test the effects of lactation status the method of mixed model was applied [27]. The variables week, lactation status, herd and age were included in the model as fixed effects and mare as random effect to account for repeated measures within the same individual. The full model was as follows:

$$y_{ijkt} = \alpha_t + \gamma_j + \iota_k + (\alpha\gamma)_{jt} + (\alpha\iota)_{ik} + (\gamma\iota)_{jk} + (\alpha\gamma\iota)_{jkt} + \beta a_{ij} + s_{ij} + \epsilon_{ijkt} \quad \text{(Eq. S2)}$$

where y_{ijt} is the haematological value for mare i at herd j in week t with lactation status k. The α_t is the effect of week, γ_j is the effect of herd and ι_k is the effect of lactation status. Interactions between the main terms were included in the model to account for that effects of one variable could depend on another variable. The term s_{ij} is the random effect of mare. The model was fitted with maximum likelihood using the lmer function in the lme4 package [28] in the statistical software R [29]. Fixed effects were tested with a likelihood ratio test and removed from the model if not significant.

Results

There were 81 lactating and 16 non-lactating mares in the herd in South Iceland, and 50 lactating and 5 non-lactating mares in the herd in North Iceland. In seven cases it was not known whether a mare had been lactating as this information was gathered afterwards. Therefore, the analysis was based on 153 mares.

The results showed that there was a significant interaction between week and lactation status (p<0.001) and between week and herd (p<0.001) for RBC. This indicates that the change between weeks was different depending on lactation status and for the two different herds (Figure S2). For Hct the three-way interaction terms were significant (p=0.049). Three-way interactions are difficult to interpret. The estimated mean of Hct was lower for lactating mares than non-lactating mares in the first two weeks of the harvesting season and in the two recovery weeks for the herd in the north but for the herd in the south there was not much difference except three weeks after the last harvesting when the lactating mares had a higher estimated mean than non-lactating mares. This was the other way around for the herd in the north. There were only five non-lactating mares in the herd in the north and therefore these results should be interpreted with that in mind. There was a significant interaction between week and lactation status (p<0.004) and between week and herd (p<0.001) for Hgb. These interactions were also significant for MCV (p<0.001), and for MCH the three-way interaction was significant (p=0.011). The interaction between herd and lactation status was significant for MHCH (p=0.039) and between week and herd (p<0.001). For RDW the interaction between week and lactations status was significant (p=0.003) and the interactions between week and herd (p<0.001).

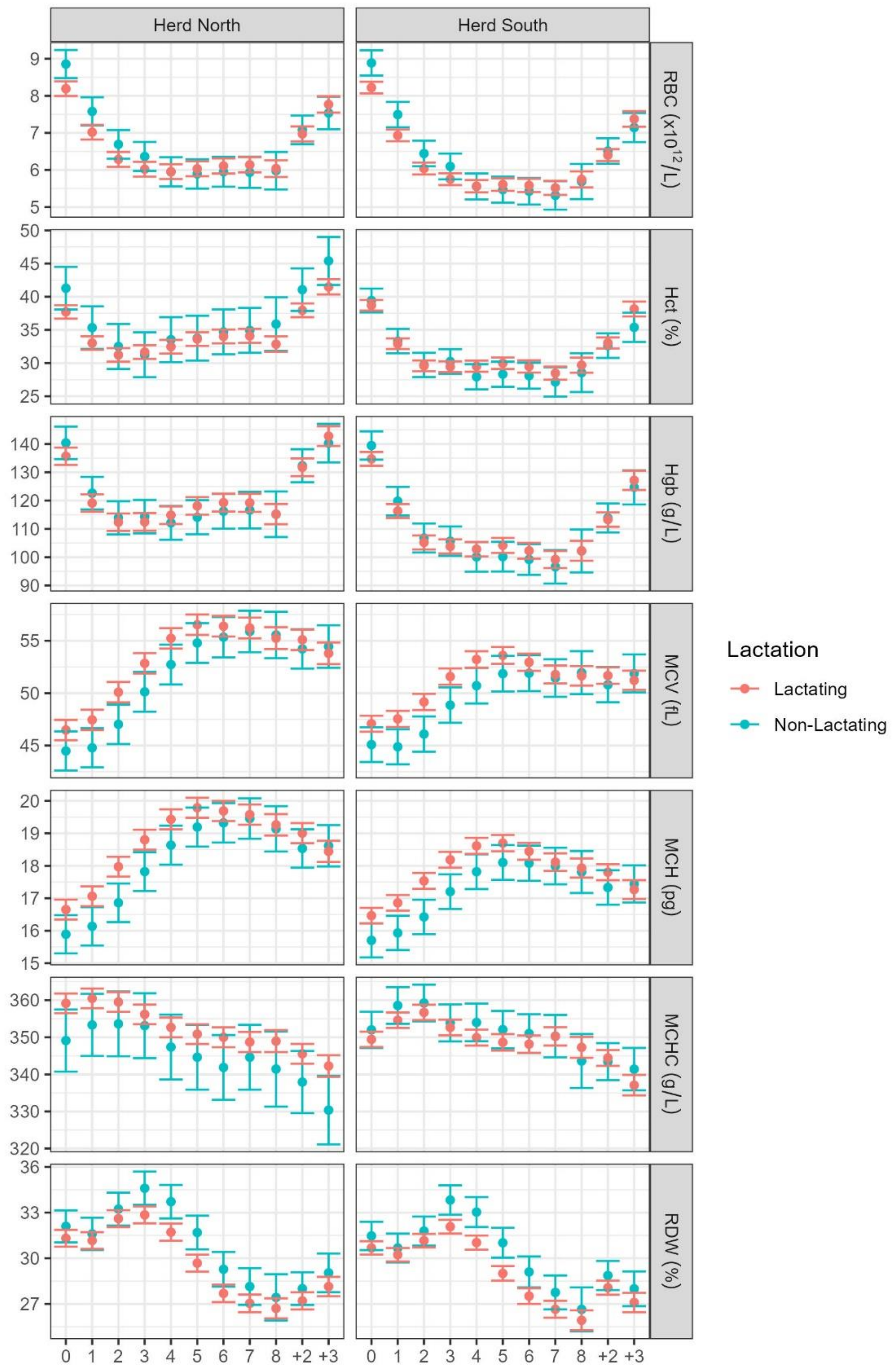

Figure S2. Estimated mean of the haematological variables for each herd, lactation status and average age. From the final models (Eq. S2) for each week of the season: just before the first blood harvesting took place (0), and then weekly during the harvesting season, with the numbers 1-8 representing the number of blood harvesting occasions preceding the analysed sample. The last two sampling points represent two and three weeks after the last blood harvesting (+2,+3). The vertical lines show the 95% confidence intervals. RBC: red blood cell count, Hct: haematocrit, Hgb: haemoglobin, MCV: Mean corpuscular volume, MCHC: mean corpuscular haemoglobin concentration, MCH: mean corpuscular haemoglobin, RDW: red cell distribution width.
