# Supplementary material for: The Effect of Repeated Blood Harvesting from Pregnant Mares on Haematological Variables
Source: Animals (Basel). 2024 Feb 28;14(5):745. doi: 10.3390/ani14050745 (PMC10931342; doi:10.3390/ani14050745)
Supplement: Supplementary file 1 [file animals-14-00745-s001.zip › animals-2867686 Supplementary Table S1 Rev1.pdf]

Supplementary Materials

Table S1. Descriptive statistics for the haematological variables by weeks: first week of the season, just before the first blood harvesting took place (0), one week after the 1st to the 8th blood harvesting (1-8) and 2 and 3 weeks after the last blood harvesting (+2,+3).

Variable	Week	N	Min	Mean	SD	Max
RBC	0	159	5.83	8.32	0.96	11.44
RBC	1	156	5.11	7.03	0.83	9.98
RBC	2	152	3.93	6.17	0.73	8.06
RBC	3	146	4.05	5.86	0.74	7.92
RBC	4	137	3.89	5.68	0.68	7.75
RBC	5	127	4.38	5.73	0.71	7.81
RBC	6	109	4.22	5.76	0.72	7.7
RBC	7	92	4.24	5.77	0.77	7.93
RBC	8	57	4.53	5.79	0.64	7.81
RBC	+2	138	5.01	6.64	0.8	8.76
RBC	+3	64	4.34	7.46	0.79	8.81
HCT	0	159	26.2	38.64	4.32	52.5
HCT	1	156	22.9	33.01	3.77	43.9
HCT	2	152	20.9	30.23	3.52	40.3
HCT	3	146	21.2	30.16	3.66	39.8
HCT	4	137	19.9	30.45	3.7	39.4
HCT	5	127	21.5	31.22	4.19	40.9
HCT	6	109	21.4	31.42	4.33	41.2
HCT	7	92	23.7	31.36	4.47	41.3
HCT	8	57	25.7	31.25	3.4	39.8
HCT	+2	138	25.5	35.15	4.52	45.4
HCT	+3	64	26	39.6	4.47	48.3
HGB	0	159	99	136	13	179
HGB	1	156	88	118	12	149
HGB	2	152	77	108	12	137
HGB	3	146	81	107	12	136
HGB	4	137	74	107	12	132
HGB	5	127	79	109	14	140
HGB	6	109	76	109	14	145
HGB	7	92	82	109	15	142
HGB	8	57	90	108	11	135
HGB	+2	138	91	121	14	154
HGB	+3	64	89	134	15	163
MCV	0	159	37.5	46.6	3.4	55.2
MCV	1	156	36.8	47.1	3.6	57.4
MCV	2	152	38.2	49.1	3.9	60.9
MCV	3	146	41.2	51.7	4	63.4

MCV	4	137	41.8	53.8	4.2	65.8
MCV	5	127	42.9	54.6	4.4	66.5
MCV	6	109	44.2	54.6	4.5	66.1
MCV	7	92	43.7	54.5	4.7	64.5
MCV	8	57	43.3	54.2	4.3	62
MCV	+2	138	43.2	53	4.3	62.6
MCV	+3	64	42.2	53	3.7	60.8
MCH	0	159	13.5	16.4	1.1	19.2
MCH	1	156	13.6	16.8	1.2	19.9
MCH	2	152	14.1	17.6	1.3	21.5
MCH	3	146	14.9	18.3	1.3	22
MCH	4	137	15.4	18.9	1.3	22.1
MCH	5	127	15.7	19.1	1.4	22.5
MCH	6	109	15.8	19	1.4	22.6
MCH	7	92	15	19	1.5	22.4
MCH	8	57	15.3	18.8	1.4	21.9
MCH	+2	138	14.7	18.2	1.4	21.8
MCH	+3	64	15.1	18	1.2	20.5
MCHC	0	159	320	353	11	378
MCHC	1	156	326	357	11	384
MCHC	2	152	331	358	9	379
MCHC	3	146	332	354	9	382
MCHC	4	137	321	351	10	372
MCHC	5	127	325	350	9	372
MCHC	6	109	325	349	9	371
MCHC	7	92	327	348	8	365
MCHC	8	57	324	348	9	369
MCHC	+2	138	315	344	9	367
MCHC	+3	64	308	339	10	357
RDW	0	159	26.3	31	2.2	37.4
RDW	1	156	25.3	30.6	2.3	37.2
RDW	2	152	26.6	31.8	2.7	38.8
RDW	3	146	27.4	32.6	2.5	39.7
RDW	4	137	25.8	31.7	2.4	39.8
RDW	5	127	25.7	29.7	2.1	37
RDW	6	109	24.3	28	1.7	35.2
RDW	7	92	23.5	27.3	1.7	33.4
RDW	8	57	23.4	26.4	1.4	30.9
RDW	+2	138	22.9	27.8	1.9	35.5
RDW	+3	64	25.1	28	1.4	31.3
WBC	0	159	5.21	9.5	1.73	16.22
WBC	1	156	5.68	9.8	1.73	16.66

WBC	2	152	5.57	9.63	1.81	15.35
WBC	3	146	5.5	8.99	1.68	15.32
WBC	4	137	6.16	8.84	1.53	13.54
WBC	5	127	5.96	8.61	1.48	15.2
WBC	6	108	5.08	8.63	1.46	13.48
WBC	7	92	5.78	8.57	1.36	13.69
WBC	8	57	5.96	8.54	1.54	13
WBC	+2	138	6.2	8.51	1.36	12.2
WBC	+3	64	5.77	8.73	1.38	11.93
NEU	0	159	1.8	4.47	0.87	7.27
NEU	1	156	2.65	4.78	0.96	8.58
NEU	2	152	2.34	4.74	1.09	10.53
NEU	3	146	2.65	4.47	0.94	8.04
NEU	4	137	2.08	4.48	0.88	6.94
NEU	5	127	2.63	4.42	0.89	9.46
NEU	6	108	2.47	4.47	0.77	6.76
NEU	7	92	3.07	4.6	0.76	7.53
NEU	8	57	3.45	4.7	0.89	7.26
NEU	+2	138	2.53	4.46	0.78	7.28
NEU	+3	64	2.82	4.7	0.79	6.46
LYM	0	159	1.48	3.53	1.02	7.79
LYM	1	156	1.58	3.49	1	7.52
LYM	2	152	1.69	3.38	0.89	6.62
LYM	3	146	1.26	3.17	0.89	7.28
LYM	4	137	1.28	3.12	0.88	6.7
LYM	5	127	1.31	3.06	0.94	7.17
LYM	6	108	1.35	3	0.88	6.98
LYM	7	92	1.01	2.84	0.91	7.4
LYM	8	57	1.46	2.71	0.82	6.22
LYM	+2	138	1.39	2.91	0.86	5.91
LYM	+3	64	1.06	2.88	0.91	5.61
MONO	0	159	0.27	0.57	0.13	1.11
MONO	1	156	0.32	0.61	0.14	1
MONO	2	152	0.29	0.6	0.15	1.29
MONO	3	146	0.29	0.54	0.14	1.08
MONO	4	137	0.2	0.55	0.14	1.08
MONO	5	127	0.33	0.53	0.12	0.97
MONO	6	108	0.29	0.52	0.13	0.93
MONO	7	92	0.31	0.52	0.13	0.98
MONO	8	57	0.25	0.51	0.14	1.16
MONO	+2	138	0.25	0.47	0.13	0.92
MONO	+3	64	0.33	0.52	0.1	0.81

EOS	0	159	0.18	0.85	0.46	3.13
EOS	1	156	0.23	0.82	0.4	2.28
EOS	2	152	0.13	0.84	0.49	2.44
EOS	3	146	0.12	0.73	0.45	2.35
EOS	4	137	0.14	0.62	0.38	2.55
EOS	5	127	0.17	0.54	0.28	1.81
EOS	6	108	0.15	0.56	0.28	1.71
EOS	7	92	0.07	0.54	0.27	1.52
EOS	8	57	0.24	0.57	0.26	1.5
EOS	+2	138	0.16	0.58	0.29	1.86
EOS	+3	64	0.22	0.57	0.26	1.37
BASO	0	159	0.01	0.07	0.07	0.71
BASO	1	156	0.01	0.09	0.07	0.5
BASO	2	152	0	0.07	0.05	0.29
BASO	3	146	0	0.07	0.06	0.32
BASO	4	137	0.01	0.07	0.06	0.31
BASO	5	127	0.01	0.06	0.05	0.21
BASO	6	108	0.01	0.07	0.05	0.32
BASO	7	92	0.01	0.08	0.04	0.2
BASO	8	57	0.01	0.06	0.04	0.15
BASO	+2	138	0	0.08	0.05	0.37
BASO	+3	64	0.02	0.07	0.03	0.17
